# Estimating the COVID-Related Deaths Attributable to President Trump’s Early Pronouncements About Masks

**DOI:** 10.1177/0020731420960345

**Published:** 2020-09-23

**Authors:** Robert A. Hahn

**Affiliations:** 1 Department of Anthropology, Emory University, Atlanta, Georgia, USA

**Keywords:** presidential pronouncements, COVID-19, masks, attributable mortality

## Abstract

The goal of this analysis is to estimate the proportion of COVID-19 deaths attributable to President Donald Trump’s early pronouncements about voluntary mask use and his intention not to use masks. Data from available research were used to estimate parameters for the calculation of population attributable risk for COVID-19 deaths reported to date. Assuming Trump’s pronouncement to have caused 25%, 50%, and 75% of the non-use of masks, estimates of Trump-attributable COVID-19 deaths to date would be, respectively, 4,244, 8,356, or 12,202. The effects of presidential pronouncements on health-related matters may have large public health consequences. Pronouncements of national leaders should be based on the best available science.

On April 3, 2020, President Donald Trump made the following pronouncement about the use of masks for preventing infection with SARS-CoV-2: “It is going to be a voluntary thing. You can do it. You don’t have to do it. I am choosing not to do it.” Here we consider the question, “What is the responsibility – the causal impact – of President Trump’s pronouncement regarding the use of masks to protect against COVID-19?” If the president says the use of masks is voluntary and that he will not use one, to what extent is he the cause of deaths that result from the non-use of masks? The effect of the president’s pronouncements may work either directly on those who hear them or indirectly through the media or social networks, which then affect the actions of their members. The COVID-19 deaths can be deaths of either the mask non-users themselves, who contract infection because they are not wearing a mask, or deaths of others whom infected mask non-users subsequently expose. On July 21, President Trump reversed his position, recommending mask use and announcing that he would use a mask. We examine the potential effects of his statements between April 3 and July 21.

We use the standard epidemiological method of population attributable risk (PAR), also called etiologic fraction, which estimates the proportion or amount of an outcome attributable to a given cause (https://activepi.herokuapp.com/courses/active-epi-course). Estimation requires assumptions that can be varied to determine their effects on the resultant hypothetical conclusions.

Many citizens respect President Trump’s authority. A CBS News Poll on March 21–23 found that 90% of Republicans trusted the president to give accurate information about the SARS-CoV-2 virus, together with 14% of Democrats (YouGov CBS News Poll). A month later, another survey reported similar results: 86% of Republicans were “very” or “somewhat likely” to trust the president to handle the pandemic effectively, along with 10% of Democrats. (The Conversation, “Coronavirus: New Survey Shows How Republicans and Democrats Are Responding Differently,” May 12.)

## Methods

Estimation of the number of deaths attributable to Trump’s mask pronouncements requires several component estimates:

1. The number of COVID-19 deaths reported in the nation between April 3 and July 21, **D_R_**
2. The COVID-19 fatality rate per SARS-CoV-2 infection, **F_I_**, is the proportion of those infected with the SARS-CoV-2 virus who die from COVID-19
3. The proportion of the population that rarely or never uses masks in public settings, **M_N_**
4. The proportion of mask rare or non-users, **T**, who are responding to Trump’s pronouncement
5. The relative risk of SARS-CoV-2 infection associated with non-use of masks, compared with the risk of infection with use of masks, **RR_M_**

We first report estimates of COVID-19 deaths that had occurred by July 21 and subtract deaths that had occurred prior to April 3.

We then estimate proportion of infections attributable to Trump’s mask pronouncements, using 3 hypothetical proportions of mask non/rare use that might have been caused by the president’s pronouncements – i.e., T = 0.25, T = 0.50, and T = 0.75. We apply these proportions in the calculation of PAR = P_e_ (RR–1)/((P_e_ (RR–1)) + 1), where P_e_ is the proportion of mask rare/non-users in the population who are responding to Trump’s pronouncements, i.e., M**_N_**× T, and RR is the relative risk of the outcome, i.e., subsequent infection, associated with mask rare/non-use, RR**_M_**, compared with mask use.

The Centers for Disease Control and Prevention (CDC) uses an existing meta-analysis^1^ to estimate the SARS-CoV-2 fatality rate, F**_I_**. Given the estimated infection fatality rate, an increase in infections associated with non-use of masks will be expected to be associated with a proportional increase in deaths. Thus, the PAR estimate of increased SARS-CoV-2 infections associated with non-mask use can be applied to estimate the number of increased deaths expected with non-mask use.

## Results

*The number of deaths reported by July 21,* D**_R_**, is 140,630 (https://www.cdc.gov/coronavirus/2019-ncov/cases-updates/us-cases-deaths.html). By April 3, 8,000 deaths had occurred. Thus, deaths that occurred between April 3 and July 21 and might be attributable to Trump’s pronouncement total 132,630.

*The infection mortality rate,* F**_I_**:

CDC bases its estimate of infection mortality rate, F**_I_**= 0.65%, on a published meta-analysis.^1^

*The proportion of the population that rarely or never uses masks in public settings,* M**_N_**:

A recent report of attitudes among U.S. adults (≥18 years of age) regarding COVID protective behaviors indicates that 17.1% report rarely or never wearing a mask in public settings.^2^

*The proportion of mask rare or non-users that are responding to Trump’s pronouncement,* T:

Because the effect of President Trump’s pronouncements on mask use is unknown, we examine a wide range of possibilities: that T = 0.25, T = 0.50, and T = 0.75 of rare and non-users refrained from mask use because of Trump’s prompting (either directly or indirectly), would not have done so otherwise, or would have done so for other reasons.

*The effectiveness of masks in preventing infection is estimated in a meta-analysis,* RR_M_:

The relative risk of SARS-CoV-2 infection using a mask compared with that of not using a mask has been estimated to be approximately 0.56,^3^ so the relative risk of infection from not using a mask is approximately 1/0.56 = 1.79.

*PAR estimates at different hypothetical levels of T*:

The PARs associated with President Trump’s mask pronouncements would be 0.032 (Estimate 1), 0.063 (Estimate 2), or 0.092 (Estimate 3), respectively, assuming Trump’s effect on mask non-use to be 0.25, 0.50, and 0.75 (Table 1). For example, the PAR for Estimate 1 (T = 0.25) is

$$\begin{array}{l} {\text{PAR}}_{\text{Estimate}\;\text{1}}\\ =0.0\text{425}\times \left(\text{1}.\text{79}-\text{1}\right)/\left(\left(0.0\text{425}\times \left(\text{1}.\text{79}-\text{1}\right)\right)+\text{1}\right)=0.0\text{32} \end{array}$$

and Estimate 1 of the number of deaths attributable to Trump’s pronouncements if 0.25 of mask non-users do not use masks because of Trump’s pronouncements is 0.032 × 132,630 = 4,244.

**Table 1.** Estimated COVID-19 Mortality Associated With President Trump’s Pronouncements on Mask Use.

Factor	Estimate 1 (CDC data, Trump effect 25%)	Estimate 2 (CDC data, Trump effect 50%)	Estimate 3 (CDC data, Trump effect 75%)
Deaths reported by July 21	132,630	132,630	132,630
Rare/non-use of masks	0.17	0.17	0.17
Trump effect on mask use (hypothesized)	0.25	0.50	0.75
Population proportion reporting rare/non-use of masks in response to Trump’s remarks	0.0425	0.085	0.1275
Relative risk of infection with mask non-use, compared with mask use	1.79	1.79	1.79
Population attributable fraction	0.032	0.063	0.092
Number of deaths to date attributable to Trump mask use pronouncements	4,244	8,356	12,202

Estimates of Trump-pronouncement-attributable COVID-19 deaths that have occurred by July 21 (Table 1) would thus be, respectively, 4,244 (Estimate 1), 8,356 (Estimate 2), or 12,202 (Estimate 3).

## Discussion

This analysis of COVID-19 deaths attributable to President Trump’s pronouncements on masks is hypothetical because it rests on assumptions that are difficult if not impossible to verify, namely the proportion of the population that rarely or never wears masks for protection from SARS-CoV-2 **because** of pronouncements made by President Trump (and would have worn masks in the absence of his pronouncements, or would not have worn masks, but for other reasons). The analysis determines the number of deaths attributable to his pronouncements **IF** 25%, 50%, or 75% of non-mask users refrained because of Trump’s statements. Although it is likely that at least some non-users were persuaded by the president’s pronouncements, the proportion actually persuaded is unknown.

The same survey that ascertained proportions of rare and non-use of masks in the population also asked about the restrictiveness of mitigation strategies and their utility in promoting safety. A percentage similar to that of rare/non-users in the population (15.6%) believed that community mitigation strategies are “too restrictive”; 25.7% of survey respondents claimed they would “feel safe if community mitigation strategies were lifted nationwide” at the time of the survey (May 5–12). The overlap of these responses with mask rare or non-use is not reported, but it may be substantial, and, if so, suggests political attitudes also allied with those expressed by the president.

The survey of reported mask non-use was conducted among person ≥17 years of age. The estimated PARs may thus apply specifically to this population. This seems reasonable insofar as only 0.03% of COVID-19 deaths have occurred among persons ≤15 years of age (https://data.cdc.gov/NCHS/Provisional-COVID-19-Death-Counts-by-Sex-Age-and-S/9bhg-hcku).

The CDC estimate of the infection mortality rate may be too high because the studies on which it is based rely on sera available for other purposes, some of which are clinical, thus possibly involving persons with health problems who may be more susceptible to SARS-CoV-2 infection. In another meta-analysis, Ioannidis uses international, randomized, population-based seroprevalence studies (denominators) and death reports (numerators) to estimate a median SARS-CoV-2 infection mortality rate (0.25%),^4^ substantially lower than the estimate of Meyerowitz.^1^ Lower estimates for the infection mortality rate would lead to lower estimated attributable mortality.

Univariate estimates of PAR often ignore other, potentially confounding causes of the same outcomes. They may thus exaggerate the estimated causal impact of single exposures. Nevertheless, the numbers of deaths possibly caused by leaders’ speech events may be large. A single death attributable to misleading information is too many.

Speech may have extensive effects on audience behavior, particularly when the speaker is a person of power and his audience is large. We do not know the power of President Trump’s voice, but, assuming a wide range of effects, we can estimate the consequences of his messages on public health outcomes. Speech can be a cause of positive health action, but also of public health harm, including death. Other public health consequences of the speech of the nation’s leaders – for example, regarding use of hydroxychloroquine or bleach, or the full opening of schools – merit similar epidemiological analysis.
